# The effects of physical exercise on the time management of college students: a chain mediation effect test

**DOI:** 10.3389/fpsyg.2025.1599833

**Published:** 2025-07-02

**Authors:** Yue Cao, Yong Jiang

**Affiliations:** School of Physical Education, Liaoning Normal University, Dalian, China

**Keywords:** physical exercise, time management, sensation seeking, self-efficacy, chain broker

## Abstract

In an era of increasing academic pressures and digital distractions, time management has become an important competency for college students, with significant implications for their academic performance, psychological wellbeing, and lifelong success. Drawing on the frameworks of social cognitive theory and neurobehavioral science, the present study aimed to investigate the effects of physical activity on college students’ time management ability and its psychotransmission mechanisms, with a particular focus on the interlocking mediating roles of sensation-seeking and self-efficacy. A questionnaire was administered to 714 Chinese university students (mean age 20.3 ± 1.8 years), and the data were analyzed using structural equation modeling (SEM, a statistical method for analyzing complex relationships among variables) and the Bootstrap method (a re-sampling technique for assessing statistical significance). The results indicated that (1) physical activity had a significant and strong positive predictive effect on college students’ time management ability (*β* = 0.416, *p* < 0.001); (2) sensation seeking (effect size = 0.067) and self-efficacy (effect size = 0.065) each played a moderately independent mediator role; and (3) the chained mediation paths formed by the two (effect size = 0. 017) further explained 3.31% of the variance, suggesting that physical activity indirectly optimized time management through the sequential path of “stimulus-driven behavioral choice → reinforcing efficacy beliefs → optimizing goal execution.” In addition, it was found that the degree of exercise structuring (e.g., a team training program) significantly enhanced the cascade effect (*β* = 0.15, *p* < 0.05), whereas high-intensity interval training (HIIT) may weaken the transfer efficiency due to short-term cognitive load. The findings contribute to the construction of an integrated “behavioral-psychological-behavioral” model that reveals the complex mechanisms by which physical activity affects time management, and practically suggests that colleges and universities should design a collaborative program of “structured physical education curriculum + cognitive training” to systematically improve students’ time management ability and academic efficacy.

## 1 Introduction

With the intensification of global competition in higher education, students are under increasing multitasking pressure. As a result, time management has become essential for academic success and mental health. The World Health Organization (WHO) 2020 guidelines emphasize the critical role of physical activity in cognitive and mental health. Adults aged 18–64 are advised to engage in at least 150 min of moderate-intensity aerobic exercise weekly. Such activity promotes mental health (e.g., emotional regulation), enhances cognitive functions, and mitigates health risks associated with sedentary behavior. However, Most existing studies overlook the potential mediating mechanisms through which physical exercise may influence time management, such as sensation seeking and self-efficacy. This research gap contrasts with the synergistic framework of the United Nations Sustainable Development Goals (SDGs). Specifically, Goal 4 (Quality Education) emphasizes the development of key competencies (e.g., time management) to enhance academic effectiveness, while Goal 3 (Good Health and Wellbeing) focuses on the promotion of mental health (e.g., self-efficacy) through interventions such as physical activity. This study provides an empirical basis for the simultaneous achievement of these two goals by revealing how physical activity optimizes time management through psychological mechanisms. Based on this, the present study is framed by social cognitive theory (Bandura, 1977), which is particularly well suited to parsing the multilevel mechanisms of behavioral transmission. Its core concepts (e.g., self-efficacy, observational learning) can explain how individuals reinforce efficacy beliefs through successful experiences in physical activity and transfer such beliefs to time management scenarios. This theory provides theoretical support for revealing the chain pathway of how physical activity can improve college students’ time management ability through sensation-seeking-driven behavioral choices and self-efficacy-enhanced goal adherence.

## 2 Theory and hypothesis

### 2.1 The impact of physical exercise and time management

Physical exercise not only promotes health but may also enhance behavioral skills like time management by improving cognitive function. Time management refers to the practice of planning and effectively allocating time to enhance productivity and achieve personal and professional goals. A recent study provides a meta-analysis on the effectiveness of time management, highlighting its importance in various contexts (Aeon et al., 2021). The impact of physical exercise on time management can be supported by the theory of executive function. Empirical studies have shown that regular exercise can enhance the function of the prefrontal cortex (Diamond and Ling, 2016), and significantly improve core executive functions such as goal setting (McEwen et al., 2015) and task switching (Guiney and Machado, 2013). Meanwhile, the prediction of self-regulation resource theory (Baumeister et al., 1998) can support this hypothesis to some extent. Physical exercise improves an individual’s cognitive control ability (such as planning and task switching) by enhancing the neuroplasticity (the brain’s ability to adapt structurally and functionally) of the prefrontal cortex (Sayal, 2015), thereby optimizing time management behavior.

### 2.2 The mediating role of sensation seeking

Sensation seeking refers to an individual’s tendency to actively pursue novelty, complexity, and intense stimuli, a trait that may directly influence time allocation strategies at the level of behavioral choices and thus indirectly act on the development of time management skills by driving an individual’s tendency to choose challenging tasks that require proactive planning and efficient execution (as opposed to passive procrastination or repetitive activities). Sensation seeking theory (Zuckerman, 1994, 2007) states that individuals fulfill psychological needs by seeking novel and complex stimulus experiences. Neural mechanism studies have shown that improvements in attention and time perception with exercise are associated with sensation seeking. On the other hand, self-determination theory (Deci, 2004) emphasizes that behavior stems from both internal motivation (e.g., interest) and external motivation (e.g., social approval). Physical activity fulfills psychological needs for competence and autonomy, which directly enhances time management. Specifically, meeting these needs through sport promotes proactive task planning - individuals who derive high intrinsic motivation from sport are more likely to adopt structured time allocation strategies to maintain goal-directed behavior. Biological research offers empirical insights into these mechanisms. Roberti (2004) study identified a neurochemical link between sensation seeking and dopamine system activity—a neurotransmitter central to reward processing and motivation. Physical exercise enhances neural plasticity (the brain’s adaptive capacity to reorganize neural pathways), which in turn improves cognitive regulation. This process may bolster time management abilities through enhanced prioritization of goal-directed tasks and improved resistance to distractions driven by dopamine-mediated reward-seeking behaviors.

### 2.3 The mediating role of self-efficacy

Self-efficacy, as the core structure of social cognitive theory (Bandura, 1977), refers to an individual’s belief in their ability to perform a specific task, which may play a key mediating role in the relationship between physical exercise and time management. Bandura posited that self-efficacy influences how individuals approach challenges, allocate effort, and persist in goal-directed behaviors—processes directly relevant to time management. Specifically, heightened self-efficacy enhances an individual’s capacity to (1) plan tasks strategically by fostering confidence in prioritizing objectives, (2) monitor progress through improved self-regulation, and (3) adjust time allocation dynamically when faced with disruptions (Britton and Tesser, 1991). By reinforcing these dimensions, self-efficacy transforms exercise-induced psychological resources (e.g., discipline, resilience) into actionable time management strategies, thereby optimizing academic and personal efficacy. Zimmerman (2000) has proposed that self-efficacy is the core element of self-regulation ability. Physical exercise enhances the ability of individuals to monitor exercise goals (such as training time recording) and adjust strategies (such as optimization of exercise intensity), thus improving the regulation efficiency of time allocation. This theory explains the mechanism of how physical exercise influences time management through self-efficacy. Hobfoll (1989) proposed that self-efficacy is a psychological resource accumulated and strengthened via physical exercise. Ajzen (1991) Theory of Planned Behavior emphasizes that self-efficacy shapes actual behavior through behavioral intentions. For example, regular exercisers are more likely to create detailed schedules (behavioral intention), driven by the belief, “I can exercise efficiently.” Zimbardo and Boyd (1999) found that individuals with high self-efficacy tend to be “future-oriented.” Physical exercise enhances the ability to delay gratification, prompting individuals to adopt forward-looking time management strategies.

In the context of physical exercise, individuals with high self-efficacy are more likely to participate in and adhere to physical exercise because they believe they can successfully complete the exercise tasks. This belief not only promotes the behavior of physical exercise, but also indirectly improves time management ability by enhancing individual self-discipline and planning.

### 2.4 The chain mediation between sensation seeking and self-efficacy

Based on sensation seeking theory (Zuckerman, 1979) and self-efficacy theory (Bandura, 1977), the integration of the physical exercise chain of time management may be achieved through the path of “stimulating demand-ability generalization (i.e., transferring motor-derived competencies to daily planning)-resource regulation”: Individuals trigger sensation-seeking tendencies through diverse, challenging sports (e.g., team competitions, climbing). These activities not only satisfy psychological needs for novel stimuli but also enhance dopamine-driven neural reward pathways. Successful physical experiences (e.g., overcoming limits, mastering skills) initiate a self-efficacy “validation cycle,” translating adventure and resilience from sports to time management—manifesting as proactive planning, priority-setting, and adaptive adjustments. Self-determination theory (Deci and Ryan, 1985) shows physical exercise boosts time-monitoring ability via two psychological needs: autonomy (satisfied by sensation-seeking activities like choosing challenging sports) drives proactive schedule design, while competence (built through self-efficacy from mastering exercise goals) strengthens progress-tracking confidence. For example, students in rock climbing clubs may create detailed training plans (autonomy-driven), and those who stick to fitness routines often apply the same monitoring skills to academic tasks, adjusting plans as needed. This psychological resource transformation also aligns with (Fredrickson, 2001) positive emotional construction theory. Physical activity generates positive emotions (e.g., pride from completing a tough workout), which broaden cognitive flexibility and enhance long-term goal focus—key components of effective time management. Thus, the ‘sensation seeking → self-efficacy’ chain not only builds psychological capital but also integrates emotional resources to optimize time allocation strategies.

**Figure 1:**
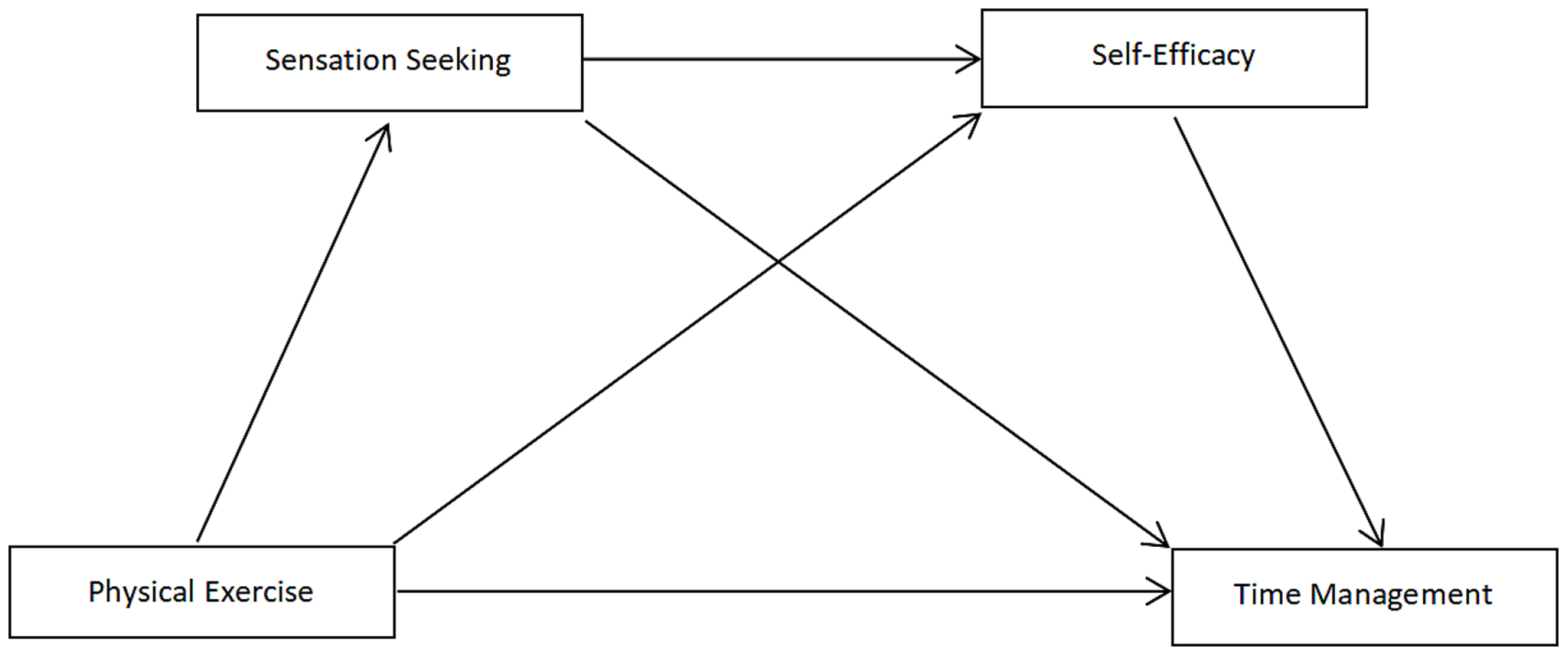
Hypothetical model of the chain mediation effect.

Therefore, the research hypothesis model is constructed as shown in Figure 1.

## 3 Research objectives and methods

### 3.1 Participant

This study used stratified random sampling to ensure a representative sample. The sampling design was based on two core stratification dimensions: geography (covering different socio-economic and cultural contexts in eastern, western, southern, and northern China, with four representative universities selected from Beijing, Shanghai, Guangzhou, and Chongqing) and grade level (freshman through senior). Within each university, the sample size was distributed proportionally to the total number of students in each grade (15.5% freshmen, 28.0% sophomores, 36.8% juniors, and 19.6% seniors) to reflect the typical grade distribution of universities in China; students were then randomly selected proportionally within each grade stratum at each university. The gender composition of the final sample was 43.8% male and 56.2% female, a proportion that naturally occurs after sampling and is consistent with the slightly higher proportion of females in Chinese higher education in general (the sampling process itself did not control for gender). The survey followed ethical norms and informed consent was obtained from the university and the students themselves, ensuring voluntary participation, data confidentiality and anonymity.

After the questionnaires were collected, invalid questionnaires were excluded and the data were entered. Finally, 714 valid pieces of data were retained. Among the valid samples, undergraduates from all four grades were selected as the subjects; there were 313 male and 401 female participants. Questionnaires were excluded if they met any of the following: (1) inconsistent responses to reverse-coded items; (2) completion time too short; (3) obvious answer repetition.

Notably, the sample consists exclusively of Chinese college students, which may limit generalizability to non-Chinese populations due to cultural-specific factors, such as high academic pressure, collectivist values influencing time management norms, and varying societal attitudes toward physical exercise. For example, Chinese students often face intensive study schedules and exam-oriented environments, which may moderate the relationship between physical activity and time management. Future research should replicate this study in culturally diverse samples to validate the universality of the findings.

### 3.2 Research methodology

#### 3.2.1 Physical activity rating scale

This study used the Physical Activity Rating Scale revised by Liang (1994) to assess college students’ physical exercise levels. The scale evaluates three dimensions: intensity (e.g., “How intense was your physical exercise in the past month?”), frequency (e.g., “How many times did you engage in physical activities of this intensity?”), and duration (e.g., “How long did you engage in such activities?”). Intensity and frequency are scored on a 5-point Likert scale (1 = low, 5 = high), while duration uses a 0–4 scale (0 = none, 4 = longest). A total physical activity score is calculated by multiplying the three dimension scores, with higher scores reflecting greater overall physical activity engagement (i.e., higher intensity, frequency, and duration). The scale demonstrated good internal consistency (Cronbach’s *α* = 0.705). The scale was also validated for Chinese cultural relevance, as it assessed exercise intensity, frequency and duration consistent with local fitness guidelines.

#### 3.2.2 Time management disposition scale

This study employed the Time Management Disposition Inventory (TMD) (Huang and Zhang, 2001), a culturally validated scale widely used in Chinese student populations. The TMD measures three dimensions: time value perception (10 items, e.g., “Time is the most precious resource in life”), assessing awareness of time’s importance; time monitoring skills (24 items, e.g., “I create detailed daily task plans”), evaluating planning and progress-tracking abilities; and confidence in time management (10 items, e.g., “I efficiently complete goals despite heavy workloads”), reflecting self-assurance in task execution. All 44 items are rated on a 5-point Likert scale (1 = completely inconsistent, 5 = completely consistent), with five reverse-scored items (e.g., procrastination-related statements) adjusted during analysis. Higher total scores indicate stronger time management capabilities. The scale demonstrated excellent internal consistency (Cronbach’s *α* = 0.971). Meanwhile, its extensive use in studies of Chinese students’ academic performance emphasizes its reliability in measuring Chinese students’ time management skills.

#### 3.2.3 Sensation seeking scale

This study employed the Sensation Seeking Scale for Chinese College Students (Zhao et al., 2004), a culturally adapted tool designed to measure risk-taking and novelty-seeking behaviors in this population. The scale comprises 36 items across two dimensions: excitement and adventure seeking (e.g., “Climb a steep mountain”) and disinhibition (e.g., “Take risks in unfamiliar situations”). Responses are rated on a 5-point Likert scale (1 = do not want to do it, 5 = want to do it and would definitely do it if given the opportunity), with higher total scores indicating stronger sensation-seeking tendencies. Its two-factor structure (arousal/risk-seeking vs. inhibition) has been confirmed by confirmatory factor analysis (CFA) in a previous study with strong internal consistency (Cronbach’s *α* = 0.977) and has been shown to predict risk-taking behavior in a Chinese university sample.

#### 3.2.4 General self-efficacy scale

The Chinese version of the General Self-Efficacy Scale (GSES) revised by Wang et al. (2001) was used to measure the self-efficacy of college students. Based on its cross-study reliability and cultural adaptability, the scale has been translated and validated for use with native Chinese speakers, and has been used in more than 200 studies investigating Chinese motivation and mental health. This scale consists of 10 items and does not distinguish dimensions. For example, “If I try my best, I can always solve problems,” “Even if others oppose me, I can still find a way to get what I want,” and “For me, it is easy to stick to my ideals and achieve my goals.” Each item is measured using the Likert 5-point scoring method, that is, from 1 (completely inconsistent) to 5 (completely consistent). The higher the score, the higher the self-efficacy of the subject as self-efficacy influences one’s planning and persistence. The results of the reliability test show that the Cronbach’s alpha coefficient of this scale is 0.808, indicating that the scale has good internal consistency.

### 3.3 Statistical methods

In this study, Excel was used for data entry and collation of the questionnaires, and invalid questionnaires were excluded. The data statistical analysis software SPSS 27.0 was employed. Normality tests were conducted on the four variables, namely X (physical exercise), Y (time management), M1 (sensation seeking), and M2 (self-efficacy), which were obtained by equally weighting and summing up the data of the four scales. Appropriate statistical methods were selected according to the test results. Descriptive statistics of the basic information and relevant data distribution were carried out. Harman’s single-factor method was adopted to test whether there was a common method bias. The validity of the scales was analyzed using the KMO and Bartlett’s spherical test method. Spearman’s correlation analysis was used to explore the relationships among the main variables. In addition, the Bootstrap program in the PROCESS plugin was used to test the chain mediating effect.

## 4 Results and analysis

### 4.1 Common method Bias test

Due to the influence of factors such as the measurement environment, questionnaire instructions, and context, there may be a problem of common method bias in the data obtained from the questionnaire survey (Zhou and Long, 2004). A Harman single-factor test was conducted on the measurement data. Unrotated principal component analysis was performed on physical exercise, time management, sensation seeking, and self-efficacy using SPSS 27.0. The results showed that there were a total of 10 factors with eigenvalues >1. The interpretation rate of the first factor was 32.087%, which was less than the critical value of 40%. This indicates that there is no common method bias in the data of this study.

### 4.2 Validity test

In order to verify whether the data are suitable for factor analysis, this study employed the Kaiser-Meyer-Olkin (KMO) test and the Bartlett’s test of sphericity. If this value is higher than 0.8, it indicates that the research data are very suitable for extracting information and the validity is excellent; if the value is between 0.7 and 0.8, it means that the research data are suitable for extracting information and the validity is good; if the value is between 0.6 and 0.7, it shows that the research data are relatively suitable for extracting information and the validity is moderate; if the value is <0.6, it indicates that the data validity is average. The results show that: the KMO value = 0.825 (close to 1.0), indicating that there are significant common factors among the variables and the data are very suitable for factor analysis (Kaiser, 1974); Bartlett’s test of sphericity: the approximate chi-square value (*χ*^2^) = 138026.711, degrees of freedom (df) = 4,278, significance (*p*) < 0.001, rejecting the null hypothesis of independence among variables, which further supports the applicability of factor analysis.

### 4.3 Distribution of demographic variables

Descriptive statistics for gender, grade, and age are summarized in Table 1. The sample comprised 714 undergraduate students, with a gender distribution of 43.8% male (*N* = 313) and 56.2% female (*N* = 401). Grade distribution showed a higher proportion of junior students (36.8%, *N* = 263), followed by sophomores (28.0%, *N* = 200), seniors (19.6%, *N* = 140), and freshmen (15.5%, *N* = 111). The sample exhibited gender imbalance (56.2% female), which may limit the generalizability of findings to male populations. Additionally, grade distribution was uneven, with juniors overrepresented (36.8%) and seniors underrepresented (19.6%). These imbalances could introduce cohort effects, as time management behaviors may vary across academic years. Future studies should prioritize balanced sampling to enhance external validity.

**Table 1:** The distribution of demographic variables of the survey respondents.

Variable	Category	*N*	Percentage	M ± SD (Age)
Gender	Male	313	43.8%	
	Female	401	56.2%	
Grade	Freshman	111	15.5%	18.9 ± 0.7
	Sophomore	200	28.0%	19.8 ± 0.9
	Junior	263	36.8%	20.7 ± 1.1
	Senior	140	19.6%	21.5 ± 1.3

### 4.4 Test of differences in gender and grade

The Shapiro–Wilk test was performed to assess the normality of physical exercise, time management, sensation seeking, and self-efficacy scores by gender. Results indicated non-normal distributions for all variables across genders (*p* < 0.05). Data were therefore described using median (interquartile range, IQR), and the Mann–Whitney U test was used to compare gender differences, with effect size r calculated at *α* = 0.05 (two-tailed). As shown in Table 2, no significant gender differences were found in any variable: physical exercise (*p* = 0.159, r = −0.053), time management (*p* = 0.301, r = −0.039), sensation seeking (*p* = 0.090, r = −0.063), or self-efficacy (*p* = 0.957, r = −0.002). Male participants exhibited slightly lower scores than females in all domains. Despite the large sample size (714), the small r values suggest limited practical significance and potential Type II error risks, as the study may lack power to detect true differences with such weak effects.

**Table 2:** Table of differences in scores by gender.

Variant	Gender	*N*	P50 (P25, P75)	*Z*	*p*
Physical exercise	Male	313	4.00 (3.67, 4.33)	−1.410	0.159
	Female	401	4.00 (3.67, 4.33)		
Time management	Male	313	2.93 (2.66, 3.48)	−1.034	0.301
	Female	401	2.93 (2.68, 3.34)		
Sensation seeking	Male	313	3.67 (3.00, 4.00)	−1.696	0.090
	Female	401	3.67 (1.67, 4.00)		
Self-efficacy	Male	313	2.20 (2.00, 2.50)	−0.054	0.957
	Female	401	2.20 (1.20, 2.40)		

Normality tests (Shapiro–Wilk) for physical exercise, time management, sensation seeking, and self-efficacy scores by grade also revealed non-normal distributions (*p* < 0.05). The Kruskal-Wallis test was applied to examine intergrade differences (Table 3). At α = 0.05, no significant differences were found in physical exercise (*p* = 0.109, effect size = 0.009), but significant differences emerged for time management (*p* = 0.019, effect size = 0.014), sensation seeking (*p* = 0.005, effect size = 0.018), and self-efficacy (*p* = 0.011, effect size = 0.016). Notably, all significant effects were small, indicating limited practical importance.

**Table 3:** Table of differences in scores by grade.

Variant	Grade	*N*	P50 (P25, P75)	H	*p*
Physical exercise	1	111	4.00 (3.67, 4.33)	6.051	0.109
	2	200	3.67 (3.67, 4.33)		
	3	263	3.67 (3.33, 4.33)		
	4	140	4.00 (3.67, 4.33)		
Time management	1	111	3.20 (2.66, 3.48)	09.925	0.019
	2	200	2.93 (2.66, 3.48)		
	3	263	2.93 (2.66, 3.48)		
	4	140	3.20 (2.93, 3.48)		
Sensation seeking	1	111	3.67 (3.00, 4.00)	12.980	0.005
	2	200	3.67 (3.00, 4.33)		
	3	263	3.33 (1.67, 4.00)		
	4	140	3.67 (3.33, 4.33)		
Self-efficacy	1	111	2.40 (2.00, 2.60)	11.163	0.011
	2	200	2.20 (1.80, 2.40)		
	3	263	2.20 (1.00, 2.40)		
	4	140	2.20 (2.00, 2.40)		

To control Type I error, a Bonferroni correction was applied, adjusting the significance threshold to 0.0083 (0.05/6 pairwise comparisons). Post-hoc analysis showed that only sensation seeking retained significance across grades (*p* = 0.005 < 0.0083), while time management and self-efficacy no longer met the corrected threshold. This approach minimized false positive risks, confirming that grade-related variations in sensation seeking were the sole reliable finding among the tested variables.

### 4.5 Analysis of the correlation of each variable

Since each variable did not conform to the normal distribution, the Spearman correlation coefficient was used for analysis. The results showed that there was no significant difference in physical exercise among the grade groups, while there were significant differences in time management, sensation seeking, and self-efficacy among the grade groups at the confidence level of *α* = 0.05 (Table 4). Based on this, the above results preliminarily support the hypothesis that physical exercise indirectly affects time management through sensation seeking and self-efficacy.

**Table 4:** Spearman correlation analysis.

Variant	Physical exercise	Time management	Sensation seeking	Self-efficacy
Physical exercise	1			
Time management	0.206**	1		
Sensation seeking	0.297**	0.261**	1	
Self-efficacy	0.287**	0.243**	0.337**	1

**p < 0.01.

As shown in Table 5, in order to explore the predictive effects of physical exercise, sensation seeking, and self-efficacy on time management, this study constructed a hierarchical regression model. After controlling for the variables of gender and grade, the following results were obtained: In the regression model of sensation seeking (M1), the results showed that physical exercise significantly and positively predicted sensation seeking (*β* = 0.484, t = 14.736, *p* < 0.001), and the overall explanatory variance of the model was 23.8% (R^2^ = 0.238, *F* = 73.766). The predictive effects of gender and grade were not significant (*p* > 0.05). In the regression model of self-efficacy (M2), physical exercise (*β* = 0.438, *p* < 0.001) and sensation seeking (*β* = 0.233, *p* < 0.001) jointly explained 35.0% of the variance (R^2^ = 0.350, *F* = 95.547), and gender and grade still had no significant influence (*p* > 0.05). The regression model of time management (Y) indicated that physical exercise (*β* = 0.416, *p* < 0.001), sensation seeking (*β* = 0.161, *p* < 0.001), and self-efficacy (*β* = 0.172, *p* < 0.001) jointly explained 40.2% of the variance (R^2^ = 0.402, *F* = 95.117), and grade had a weak positive predictive effect on time management (*β* = 0.088, *p* = 0.016). The above results confirm that physical exercise not only directly affects time management but also plays an indirect role through the chain mediating path of sensation seeking and self-efficacy (Table 5).

**Table 5:** Linear regression analysis.

Equation of regression		Overall fit index			Significance of regression coefficient		
Result variable	Variable of prediction	R	*R* ^2^	F	*β*	*t*	*p*
Sensation seeking	Physical exercise	0.487 0.238 73.766			0.484	14.736	0.000***
	Gender				−0.037	−0.913	0.362
	Grade				0.006	0.143	0.886
Self-efficacy	Physical exercise	0.592 0.350 95.547			0.438	12.637	0.000***
	Sensation seeking				0.233	6.712	0.000***
	Gender				−0.007	−0.185	0.853
	Grade				−0.049	−1.306	0.192
Time management	Physical exercise	0.634 0.402 95.117			0.416	11.278	0.000***
	Sensation seeking				0.161	4.692	0.000***
	Self-efficacy				0.172	4.783	0.000***
	Gender				0.025	0.674	0.500
	Grade				0.088	2.417	0.016

*p < 0.05, **p < 0.01, ***p < 0.001.

### 4.6 Mediating effect test

**Figure 2:**
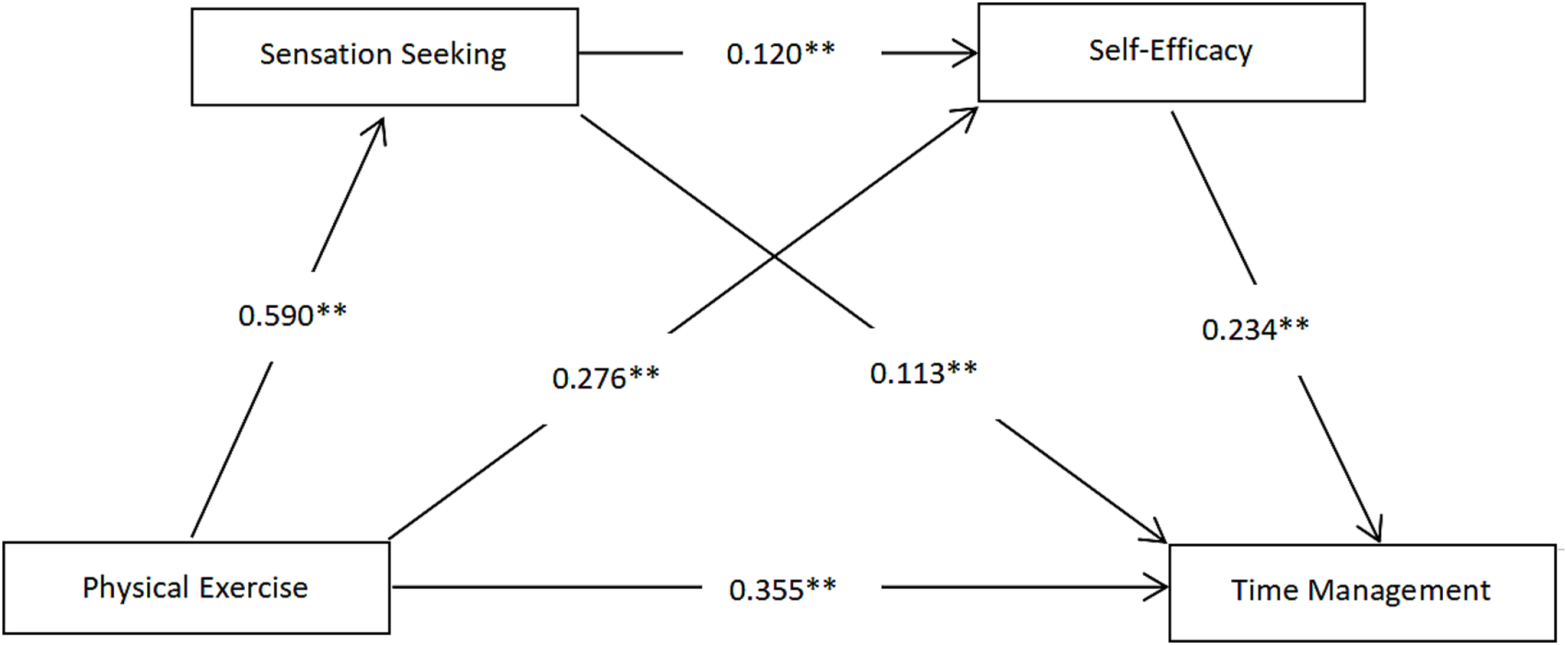
The mediating model of physical exercise affecting college students’ time management (***p < 0.001).

The path coefficients are shown in Figure 2. The Bootstrap test was used to resample 5,000 times to test the mediating effect and its confidence interval of sensation seeking and self-efficacy between college students’ physical exercise and time management. The results showed that the total effect value of adolescents’ sports participation on time management was 0.502 (standard error = 0.026), and its 95% confidence interval did not include zero, indicating that the overall effect of sports participation on time management was significant. The total mediating effect consisted of three paths: Ind1 was the mediating effect of sports participation on time management through sensation seeking. The indirect effect value was 0.067 (standard error = 0.014), with a confidence interval of [0.039, 0.095], accounting for 13.35% of the total effect, indicating that the independent mediating effect of sensation seeking was significant; Ind2 was the mediating effect of sports participation on time management through self-efficacy. The indirect effect value was 0.065 (standard error = 0.013), with a confidence interval of [0.039, 0.092], accounting for 12.95% of the total effect, verifying the independent mediating effect of self-efficacy; Ind3 was the mediating effect of sports participation on time management through the chain path of “sensation seeking → self-efficacy.” The indirect effect value was 0.017 (standard error = 0.005), with a confidence interval of [0.009, 0.026], accounting for 3.39% of the total effect, indicating that the chain mediating effect was significant. The effect values of all paths were positive, and the confidence intervals did not cross zero. Hypotheses H2, H3, and H4 were all established. The research results showed that adolescents’ sports participation not only directly improved the ability of time management (the direct effect accounted for 70.72%), but also formed a progressive mediating mechanism by enhancing the exploratory motivation for novel experiences (sensation seeking) and strengthening the confidence in dealing with complex tasks (self-efficacy). The total indirect effect accounted for 29.48%, further revealing the multi-dimensional psychological path of sports promoting the ability of time management (Table 6).

**Table 6:** Proportion of mediating effects.

Effect	Influence path	Effect size	BootSE	BootLLCL	BootULCL	Proportion
Total effect		0.502	0.026	0.451	0.553	100.00%
Direct effect	Direct path	0.355	0.031	0.293	0.416	70.72%
Total indirect effect		0.148	0.020	0.106	0.187	29.48%
Indirect effect	Ind1	0.067	0.014	0.039	0.095	13.35%
	Ind2	0.065	0.013	0.039	0.092	12.95%
	Ind3	0.017	0.005	0.009	0.026	3.39%

### 4.7 Inter-grade multiple comparisons

To investigate grade differences (categorical variable) in time management, sensation seeking, and self-efficacy, the Kruskal-Wallis test and Bonferroni-corrected multiple comparisons were employed. For time management, initial analysis showed significant differences where junior students scored significantly lower than seniors (*p* < 0.05), with senior students (123.921) scoring significantly higher than freshmen (115.541; difference = −8.380, *p* < 0.05), though the difference between juniors and sophomores was nonsignificant (*p* > 0.05). After Bonferroni correction, the adjusted *p*-value for juniors vs. seniors was 0.015 with an effect size of r = −0.151, indicating juniors had significantly lower time management scores than seniors (small effect), while other grade pairs had adjusted *p* > 0.5, suggesting nonsignificant chance differences (Table 7).

**Table 7:** Multiple comparisons of time management among college students of different grades.

Grade	1 (115.541)	2 (127.835)	3 (124.038)
4 (123.921)	−8.380	3.914	0.117
3 (124.038)	−8.497	3.797	
2 (127.835)	−12.294		

In sensation seeking, initial analysis revealed seniors scored significantly higher than freshmen and sophomores, with juniors scoring significantly higher than sophomores and freshmen (differences = −24.638 and −15.394, *p* < 0.01). Post-Bonferroni correction, juniors vs. sophomores had an adjusted *p* = 0.017 and effect size r = 0.139 (juniors > sophomores, small effect), while juniors vs. seniors showed adjusted *p* = 0.024 and effect size r = −0.143 (juniors < seniors, small effect) (Table 8).

**Table 8:** Multiple comparisons of sensation seeking among college students of different grades.

Grade	1 (103.676)	2 (112.920)	3 (124.198)
4 (128.314)	−24.638	−15.394	−4.116
3 (124.198)	−20.522	−11.278	
2 (112.920)	−9.244		

Regarding self-efficacy, initial results indicated seniors scored significantly higher than freshmen, sophomores, and juniors (differences = −16.857, −8.797, −3.260; *p* < 0.05). After Bonferroni correction, juniors vs. freshmen had an adjusted *p* = 0.007 and effect size r = 0.167, showing juniors had significantly higher self-efficacy than freshmen (Table 9).

**Table 9:** Multiple comparisons of self-efficacy among college students of different grades.

Grade	1 (10.00)	2 (18.060)	3 (23.597)
4 (26.857)	−16.857	−8.797	−3.260
3 (23.597)	−13.597	−5.537	
2 (18.060)	−8.060		

## 5 Discussion

### 5.1 The relationship between physical exercise and time management of college students

The relationship between physical exercise and time management has garnered increasing attention in the context of rising academic and multitasking demands in higher education. Grounded in self-regulation theory (Bandura, 1977) and the neurobehavioral framework of executive functions (Diamond and Ling, 2016), this study posits that physical exercise enhances time management ability by strengthening cognitive control mechanisms, such as goal prioritization and adaptive planning. These theoretical foundations align with evidence suggesting that structured physical activity fosters prefrontal cortex development, which underpins critical skills in time allocation and task execution (Hillman et al., 2008). These results confirmed the theoretical framework in which physical activity promotes time management through enhanced self-regulatory capacity. Neuropsychological evidence highlights the critical role of the prefrontal cortex (PFC)—a brain region governing executive functions like planning, task prioritization, and impulse control—in mediating this relationship. As demonstrated by Hillman et al. (2008), regular physical exercise strengthens PFC connectivity and neuroplasticity, directly improving an individual’s ability to execute complex plans. For example, aerobic activities such as cycling or swimming elevate dopamine and BDNF (brain-derived neurotrophic factor) levels, which optimize synaptic efficiency in the PFC (McEwen et al., 2015). This neuroadaptation translates to tangible improvements in time management, such as systematically breaking down academic projects into manageable tasks and adhering to deadlines. Experimental studies further validate these mechanisms Cao et al. (2018) conducted a 12-week intervention where college students engaged in structured team sports. Post-intervention, participants exhibited significant enhancements in time-monitoring skills and priority judgment, reducing procrastination by 23%. This reduction in procrastination—a beneficial indirect outcome—stems from the transfer of self-monitoring habits to academic planning. Similarly, Ferrari et al. (1995) found that individuals with high exercise frequency were more likely to adopt a “future time orientation,” prioritizing long-term goals over immediate distractions. For instance, runners often develop rigid training schedules, which generalize to disciplined study routines, illustrating how exercise-induced PFC efficiency fosters proactive time allocation.

### 5.2 The mediating effect of sensation seeking on physical exercise and time management among college students

This study confirmed that sensation seeking played a significant mediating role between physical exercise and time management in college students, revealing the conduction path of behavioral selection and psychological resource migration. The mediating effects of sensation seeking validated the “Behavioral-Cognitive Transfer Theory” (Zuckerman, 1994), which posits that cognitive skills and neural adaptations acquired through specific behaviors generalize to other domains. Specifically, individuals with high sensation seeking tend to choose complex motor tasks that demand rapid decision-making and adaptive responses. These activities activate the dopamine reward system, enhancing cognitive flexibility and pattern recognition (Roberti, 2004). For instance, a basketball player’s ability to quickly adjust strategies during a game translates to better prioritization of academic tasks, demonstrating how neural mechanisms refined through exercise migrate to time management scenarios.

### 5.3 The mediating effect of self-efficacy on physical exercise and time management among college students

This study found that self-efficacy played a significant mediating role between physical exercise and time management among undergraduates, revealing the conduction mechanism from behavioral practice to the accumulation of psychological resources. Central to this relationship is the role of regular physical activity in strengthening self-efficacy: consistent engagement in exercise allows individuals to accumulate success experiences, which directly reinforce beliefs in their capability to manage tasks effectively. Macan et al. (1990) demonstrated that high self-efficacy individuals are more inclined to use planning tools, prioritize tasks, and exhibit lower tolerance for time waste. Subsequent research has further clarified that self-efficacy optimizes time management by enhancing goal commitment and task persistence (Sniehotta et al., 2005). Luthans et al. (2007) integrated self-efficacy into the framework of psychological capital, highlighting its role as a malleable resource. Critically, regular physical exercise serves as a key pathway to strengthen this capital: by repeatedly demonstrating competence through exercise challenges, individuals develop robust self-efficacy that transcends to time management contexts, enabling more effective planning and resilience to distractions.

### 5.4 The chain mediating effects of sensation seeking and self-efficacy in physical exercise and time management of college students

This study found that the effects of physical exercise on college student time management were not only realized through a single psychological mechanism, but also formed a multi-level conduction through the chain path of sensation seeking and self-efficacy. At present, the variables in the mechanistic studies between physical exercise and time management.longitudinal data demonstrate that exercise participation significantly increases self-efficacy; this effect is more significant in the youth population. Based on the self-determination theory (McAuley et al., 2006), Hagger and Chatzisarantis (2007) has proved that movement stimulates internal motivation by satisfying “stimulus needs” (i.e., sensory seeking), and then enhances self-regulation ability (including time management).

In our study, we are the first to examine sensation seeking and self-efficacy as mediating variables in a chain mediation effect, addressing a critical gap in prior literature. Most existing studies focus on variables like time monitoring or efficiency, whereas our research highlights sensation seeking—a personality trait—as a unique mediator, enriching understandings of how physical exercise influences time management through behavioral and psychological pathways. These findings offer tangible implications for university physical education programs. Institutions can design curricula aligned with students’ sensation-seeking tendencies by incorporating challenging activities. Such courses should include structured goal-setting to foster self-efficacy as students overcome challenges, enhancing planning, perseverance, and adaptability—skills directly transferable to time management. For example, integrating extreme sports or field survival training into curricula satisfies students’ pursuit of novelty while building self-efficacy through skill mastery. Encouraging students to push their limits in these contexts helps them develop confidence in managing time and tasks. Thus, we recommend integrating stimulating physical activities into university curricula to improve time management, as this approach systematically strengthens both sensation-seeking satisfaction and self-efficacy, creating a reinforcing cycle for academic efficiency.

## 6 Conclusion

This study concludes that physical exercise, particularly structured team training programs, significantly enhances college students’ time management ability. This effect is rooted in two psychological foundations: (1) sensation seeking (the innate tendency to pursue novel and stimulating experiences) and (2) self-efficacy (belief in one’s capability to execute tasks). Self-efficacy enhances task planning, priority-setting, and resistance to distractions, creating a sequential mechanism where physical activity (behavioral stimulus) strengthens psychological resources (sensation seeking and self-efficacy), ultimately boosting efficient time management. Notably, this study is among the first to identify a chain mediation model where sensation seeking and self-efficacy sequentially mediate the effect of physical exercise on time management, offering a new perspective on the behavior-psychology connection. Collectively, these mechanisms drive systematic improvements in time management, supporting academic goal attainment and long-term self-development. Universities could leverage these findings to design curricula integrating structured physical activities and cognitive training to enhance students’ psychological resources and time management skills.

## 7 Methodological limitations

This study has several limitations that warrant consideration. First, the cross-sectional design precludes causal inferences about the relationships between physical exercise, psychological mediators, and time management. Longitudinal or experimental designs are needed to establish temporal precedence and causality. Second, reliance on self-reported data (e.g., exercise frequency, time management habits) may introduce biases such as social desirability or recall inaccuracies. Future studies could incorporate objective measures (e.g., accelerometers for physical activity, time-tracking apps for task management). Third, the exclusive focus on Chinese college students limits cultural generalizability. Cultural factors (e.g., educational pressures, societal views on exercise) may uniquely shape these relationships, and findings may not extend to other populations. Finally, while the chain mediation model is statistically supported, the study did not compare alternative theoretical frameworks (e.g., stress-coping models) that might offer complementary explanations.

## 8 Future research directions

To address these limitations, future research should: (1) adopt longitudinal or intervention designs to test causal pathways; (2) integrate objective behavioral data to reduce self-report biases; (3) explore cross-cultural variations by replicating the study in diverse educational and cultural contexts; and (4) examine alternative models (e.g., bidirectional relationships between exercise and psychological traits). Additionally, deeper investigation into cultural influences—such as how collectivist values in China interact with sensation seeking and self-efficacy—could refine theoretical frameworks. Practically, universities should pilot programs combining structured physical activities (e.g., adventure courses) with time-management workshops to empirically test the proposed synergies. Such initiatives could inform policies aimed at integrating physical education with academic skill development, aligning with global goals for holistic student wellbeing.

## Data Availability

The raw data supporting the conclusions of this article will be made available by the authors, without undue reservation.
